# An antibody that prevents serpin polymerisation acts by inducing a novel allosteric behaviour

**DOI:** 10.1042/BCJ20160159

**Published:** 2016-09-27

**Authors:** Neda Motamedi-Shad, Alistair M. Jagger, Maximilian Liedtke, Sarah V. Faull, Arjun Scott Nanda, Enrico Salvadori, Joshua L. Wort, Christopher W.M. Kay, Narinder Heyer-Chauhan, Elena Miranda, Juan Perez, Adriana Ordóñez, Imran Haq, James A. Irving, David A. Lomas

**Affiliations:** 1 Centre for Respiratory Biology, UCL Respiratory, University College London, London WC1E 6JF, U.K.; 2 Institute of Structural and Molecular Biology/Birkbeck, University of London, London WC1E 7HX, U.K.; 3 Department of Medicine, University of Cambridge, Cambridge Institute for Medical Research, Wellcome Trust/Medical Research Council Building, Hills Road, Cambridge CB2 0XY, U.K.; 4 London Centre for Nanotechnology, 17-19 Gordon Street, London WC1H 0AH, U.K.; 5 School of Biological and Chemical Sciences, Queen Mary University of London, Mile End Road, London E1 4NS, U.K.; 6 Department of Biology and Biotechnologies ‘Charles Darwin’, Sapienza University of Rome, Rome 00185, Italy; 7 Departamento de Biologia Celular, Genetica y Fisiologia, Facultad de Ciencias, Campus Teatinos, Universidad de Malaga, Malaga 29071, Spain

**Keywords:** allosteric regulation, antibodies, protein aggregation, protein conformation, protein–protein interactions, serpin

## Abstract

Serpins are important regulators of proteolytic pathways with an antiprotease activity that involves a conformational transition from a metastable to a hyperstable state. Certain mutations permit the transition to occur in the absence of a protease; when associated with an intermolecular interaction, this yields linear polymers of hyperstable serpin molecules, which accumulate at the site of synthesis. This is the basis of many pathologies termed the serpinopathies. We have previously identified a monoclonal antibody (mAb_4B12_) that, in single-chain form, blocks α_1_-antitrypsin (α_1_-AT) polymerisation in cells. Here, we describe the structural basis for this activity. The mAb_4B12_ epitope was found to encompass residues Glu32, Glu39 and His43 on helix A and Leu306 on helix I. This is not a region typically associated with the serpin mechanism of conformational change, and correspondingly the epitope was present in all tested structural forms of the protein. Antibody binding rendered β-sheet A — on the opposite face of the molecule — more liable to adopt an ‘open’ state, mediated by changes distal to the breach region and proximal to helix F. The allosteric propagation of induced changes through the molecule was evidenced by an increased rate of peptide incorporation and destabilisation of a preformed serpin–enzyme complex following mAb_4B12_ binding. These data suggest that prematurely shifting the β-sheet A equilibrium towards the ‘open’ state out of sequence with other changes suppresses polymer formation. This work identifies a region potentially exploitable for a rational design of ligands that is able to dynamically influence α_1_-AT polymerisation.

## Introduction

α_1_-Antitrypsin (α_1_-AT) is the most abundant circulating protease inhibitor and a member of the serpin superfamily. It is predominantly expressed by hepatocytes in the liver and acts to prevent excessive proteolytic damage by the enzyme neutrophil elastase, particularly in the lung [1]. In common with other inhibitory serpins, its native conformation is ‘metastable’, acting essentially as a kinetically stabilised folding intermediate. This is the basis of its mechanism of action [2]: following cleavage of a solvent-exposed ‘reactive centre loop’ (RCL) by the target protease, this loop inserts as an additional sixth strand into the central β-sheet A [3] (indicated in Supplementary Figure S1). In the process, the protease is translocated from one pole of the serpin to the other, culminating in distortion of its catalytic triad. The result is the formation of an essentially irreversible covalent complex [4].

Mutations are known that facilitate escape from the metastable conformation to achieve the thermodynamically preferred 6-stranded β-sheet A form of α_1_-AT. This is primarily associated with the formation of functionally inactive ordered aggregates known as polymers [5]. α_1_-AT is synthesised in the endoplasmic reticulum (ER) as the first step in the secretory pathway; however, polymerisation of mutant protein largely prevents further transit, resulting in accumulation in this organelle as inclusions [6]. Despite the resulting distension of the ER and changes in its luminal viscosity, α_1_-AT polymers do not trigger the unfolded protein response (UPR) [7–9]. Of many mutations in the SERPINA1 gene identified in patients presenting with plasma deficiencies [10], the Z mutation (Glu342Lys) is the most common, being present in 4% of the Northern European Caucasian population [11]. In homozygotes, it leads to the retention [6] or degradation [12] of 85–90% of the synthesised α_1_-AT [13]; the hyperaccumulation of protein is associated with liver cirrhosis [14], and the deficiency in circulating inhibitor predisposes an individual to emphysema [5]. Plasma replacement therapy is the only routine clinical treatment available. However, due in large part to high cost and concerns over efficacy [15], it is not universally offered.

The rational design of new therapies for α_1_-AT deficiency requires an understanding of the pathway through which α_1_-AT self-associates into ordered polymers. Many *in vitro* studies have found that induction of polymerisation using denaturant or heat proceeds via a polymerisation-prone intermediate ensemble [16–21], in a process that can be summarised as follows:

$$M↔M^{*}(+M^{*})\to P$$

where M represents the native monomer, M* is the activated monomeric intermediate with some characteristics of the polymer [22] and P represents the terminal, hyperstable polymer. However, it is clear that polymerisation is also a diverse process. Notably, polymers formed in the presence of denaturant lack a cryptic epitope that distinguishes them from those found *ex vivo* in patient material [23,24]. This epitope is, in contrast, expressed by polymers induced at elevated temperatures [23]. Differences are also manifest in the character of the intermediate state, which is compact when formed by heating, and expanded and molten globule-like in the presence of denaturant [25–27]. It is unsurprising then that varied models of the α_1_-AT polymer have been proposed, based on biophysical [28,29] and crystallographic [30] data. These models differ in the nature of the domain swap that forms the basis of the polymer chain, but one common feature predicted by all extant models is an expanded 6-stranded β-sheet A, with RCL residues accommodated in equivalent positions to the canonical cleaved, and therefore hyperstable, conformation (Supplementary Figure S1). This reflects the ability to block polymerisation using peptide mimetics of the RCL and the pronounced stability in denaturants [6,31]. However, the feasibility of a polymer model does not itself predicate its pathological relevance: the majority of synthesised Z α_1_-AT does not polymerise but is degraded by the ER-associated degradation pathway [32], indicating that distinct populations are present in a physiological setting.

The close relationship between conformational stability and function renders the serpin scaffold amenable to fine-tuning through intermolecular interactions with a variety of ligands, such as hormones, oligosaccharides and peptides. As a result, it has been possible to identify monoclonal antibodies with non-native-binding activities, providing tools for modulation of serpin activity and stability in novel ways [33–38]. Manipulation of the polymerisation pathway represents one therapeutic strategy for these diseases of accumulation and deficiency. For example, there is a clear relationship between the kinetic stability of the native state and the tendency to polymerise [39]. However, previous attempts at using RCL peptide analogues [40–42] and small-molecule compounds [43] have resulted in molecules that have abrogated α_1_-AT inhibitory activity.

We recently described a monoclonal antibody (mAb_4B12_) that blocks polymerisation of Z α_1_-AT, both when induced by heat *in vitro* and also during expression in a cellular model of disease, while retaining most inhibitory activity [44]. Here we have characterised its mechanism of action in detail. This was not a consequence of conformational selectivity: mAb_4B12_ displayed similar affinity for both native and inserted conformations, and blocked polymerisation of many α_1_-AT deficiency variants. Using a sparse cysteine-scanning protocol, the mAb_4B12_ epitope was localised to the vicinity of residues Glu32, His43 and Leu306 on helices A and I, with the side chain of His43 being an obligate component of the binding site. This is a region not typically associated with conformational modulation, and in apparent contradiction to a core tenet of models of serpin conformational change, when bound α_1_-AT exhibited an *enhanced* ability to incorporate RCL mimetic peptides. mAb_4B12_ was also able to destabilise the covalent complex with a model protease, trypsin, indicating local conformational change propagated to the base of the molecule from the binding site. These observations suggest that the antibody is behaving as an allosteric antagonist of polymerisation, and permit us to draw conclusions regarding the importance of the timing of β-sheet A opening to the polymerisation mechanism.

## Experimental

### Reagents

General reagents were obtained from Sigma-Aldrich or Alfa Aesar, and *Escherichia coli* growth media from Formedium, unless otherwise specified.

### Site-directed mutagenesis, recombinant expression and purification of α_1_-AT variants

Single-point mutations were designed using PrimerX (http://bioinformatics.org/primerx/) and introduced in pQE-30-based plasmids (Qiagen) containing wild-type (‘M’ allele) α_1_-AT [45] on a well-characterised Cys232Ser background (α_1_-AT_C232S_), whose behaviour is essentially indistinguishable from the Cys232 parent [39,45–49]. Site-directed mutagenesis was undertaken using the Quikchange site-directed mutagenesis kit according to the manufacturer's protocols (Agilent). Expression plasmids were transformed into XL-1 blue cells (Novagen), and recombinant proteins were expressed and purified as described previously [49,50], with the addition of 50 mM β-mercaptoethanol to single-cysteine mutants before buffer exchange into 20 mM Tris, 100 mM NaCl (pH 7.4) and storage at −80°C.

### Binding assay by sandwich ELISA

Assays were performed as recently described [39]. Briefly, plates were coated overnight with antigen-purified rabbit polyclonal anti-α_1_-AT antibody in phosphate-buffered saline (PBS), and they were washed (using 0.9% w/v sodium chloride and 0.05% v/v Tween 20), blocked with blocking buffer (PBS, 0.25% w/v bovine serum albumin, 0.05% v/v Tween 20 and 0.025% w/v sodium azide) for 2 h and washed again. Plates were incubated for 2 h at 4°C with 0.2 µg ml^−1^ of each antigen, followed by washing and incubation with one-third serial dilutions of 2 µg ml^−1^ mAb_4B12_ in PBS. The wells were then washed before incubating with rabbit anti-mouse HRP antibody (Abcam). The reaction was developed in the dark with TMB substrate solution (Sigma-Aldrich) and stopped with 1 M H_2_SO_4_, and an endpoint measurement of HRP activity at 450 nm was made using a SpectraMax M2e plate reader (Molecular Devices). All steps were performed at room temperature.

### Thermal stability assays

Fluorophore-based thermal stability assays were performed as detailed [39,49], using a 5-fold final concentration of SYPRO Orange dye solution (Life Technologies) in PBS, with a protein concentration of 0.1 mg ml^−1^ in 20 μl. Samples were heated from 25 to 95°C at a rate of 1°C min^−1^ on an Applied Biosystems 7500HT real-time PCR instrument, with fluorescence in the ROX range recorded. The descending arm of the curves following the transition was truncated once the intensity dropped 20% below the maximum. The midpoint of denaturation (*T*_m_) was determined from the optimum fit of an equation describing two-state unfolding [39,51,52]:

$$F_{T}=F_{N}+m_{N}(T-T_{m})+(F_{I}+m_{I}(T-T_{m}))\frac{e^{C\left(\left(1/T_{m}\right)-\left(1/T\right)\right)}}{1+e^{C\left(\left(1/T_{m}\right)-\left(1/T\right)\right)}}$$

where *F_T_* represents the fluorescence intensity at temperature *T*, *T*_m_ is the transition midpoint temperature, *F*_N_ and *F*_I_ are the fluorescence intensities of the native and intermediate states, respectively, and *m*_N_ and *m*_I_ reflect their temperature dependence around *T*_m_. Thermodynamic parameters were not derived from these curves due to a lack of reversibility of unfolding; instead, *T*_m_ is used as a relative measure of stability under the given experimental conditions.

### Gel-based experiments

Bis–Tris non-denaturing PAGE using 3–12% w/v acrylamide gels (Life Technologies) was used to evaluate the state of α_1_-antitrypsin polymerisation and antibody complex formation. The latter causes a decrease in α_1_-AT migration due to both the increased size of the antibody–antigen complex and an elevated net isoelectric point. Gel-based polymerisation endpoint experiments were performed as detailed [49]. Proteins were loaded at 2–4 μg per lane for staining with Coomassie blue and at 0.05–2 μg for fluorescence detection using a UV transilluminator.

### Förster resonance energy transfer-based polymerisation assay

As described recently [22], α_1_-AT_C232C_ (wild type) was labelled at the endogenous Cys232 site by incubation with a 10-fold molar excess of Alexa Fluor 488 maleimide or Alexa Fluor 594 maleimide (Life Technologies) overnight at 4°C. Following quenching with 5 mM cysteine, a 1 ml HiTrap Q sepharose column (GE Healthcare) was used to remove unconjugated label. The resulting protein was diluted to 0.1 mg ml^−1^ in PBS in the presence and absence of a 2-fold molar excess of antibody with a volume of 10 μl. Polymerisation was reported by an increase in Förster resonance energy transfer (FRET) between the donor (*λ*_ex_ = 470 nm) and acceptor dye (*λ*_em_ = 605 ± 15 nm) upon heating in a Realplex^4^ quantitative PCR instrument (Eppendorf). The ascending and initial descending components of the FRET signal were well described by an empirically determined single or double sigmoidal function in Prism (Graphpad):

$$\begin{array}{rl} & F_{t}=2(H+m_{h}(t\ln2-t_{0.5,2}))\left(\frac{w}{e^{\left(t_{0.5,1}/t\ln2\right)}+1}+\frac{\left(1-w\right)}{e^{\left(t_{0.5,2}/t\ln2\right)}+1}\right)+L \end{array}$$

where *F_t_* is the fluorescence at time *t*, *H* is the dynamic range of the curve at *t*_0.5,2_, *L* is the baseline, *m_h_* is the slope of the decay in maximal fluorescence over time, *w* is the fraction contribution to the signal by the curve with a half-time of *t*_0.5,1_ and (1 − *w*) is the fraction contribution to the signal by the curve with a half-time of *t*_0.5,2_. This equation permitted numerical calculation of the overall time to half-maximal fluorescence intensity using Octave (GNU Software Foundation).

### Polyethylene glycol conjugation experiments

Methoxypolyethylene glycol maleimide 5 kDa (PEG5K; Sigma-Aldrich) was conjugated to an introduced cysteine residue on α_1_-AT by incubating at a 10-fold molar excess to 0.5 mg ml^−1^ α_1_-AT overnight at room temperature. The reaction was quenched by the addition of a 10-fold molar excess of l-cysteine with respect to PEG5K; successful conjugation was assessed by a molecular weight shift under SDS–PAGE. For mAb_4B12_-binding assays, mAb_4B12_ and PEGylated α_1_-AT and non-PEGylated counterparts were incubated at a molar ratio of 2:1 for 30 min at room temperature in PBS. For protection assays, protein variants were first bound to mAb_4B12_ for 30 min, subsequently conjugated with PEG5K maleimide for 5 min to 3 h, and the extent of labelling was determined from a change in mobility of the antigen–antibody complex by SDS–PAGE. Steric hindrance by the PEG5K molecule of mAb_4B12_ binding was assessed by electrophoretic mobility using bis–Tris non-denaturing PAGE (Life Technologies) and ELISA assays.

### Site-directed spin labelling experiments

A free radical probe — to be used as a reporter of binding or conformational change at a specific site on α_1_-AT — was introduced onto single-cysteine mutants by incubation with 3-(2-iodoacetamido)-proxyl at a 20-fold excess in PBS overnight at 25°C. Following this, the reaction was quenched with 10 mM cysteine, and free label was removed by repurification using a 1 ml HiTrap Q sepharose column (GE Healthcare) as described previously [49]. Probe behaviour was assessed using continuous-wave electron paramagnetic resonance (CW-EPR) at room temperature on a Bruker eScan spectrometer at a protein concentration of 50–100 µM in a glass capillary, with an incident microwave power of 1 mW, a modulation amplitude of 0.1 mT, a scan range of 150 Gauss and spectra averaged over 500 scans (20 s per scan). First-derivative EPR spectra were normalised to the same number of spins using the double integral.

### Inhibitory activity assay

Inhibitory activity of α_1_-AT variants was measured and calculated as detailed previously [49,53]. Bovine trypsin (Sigma-Aldrich) was titrated using *p*′-guanidinobenzoate HCl [54]. The SI (stoichiometry of inhibition) values of wild type and mutants of α_1_-AT were determined by incubation of the protein for 15 min at room temperature with 0.1 μM bovine trypsin in 20 μl of protease assay buffer [20 mM Tris, 100 mM NaCl, 0.1% (w/v) PEG 8000, 10 mM CaCl_2_, pH 8.0]. An aliquot of 180 μl of 200 μM *N*α-benzoyl-l-arginine 4-nitroanilide substrate (Sigma-Aldrich) was added and the rate of increase in absorbance at 405 nm was recorded for 5 min using a SpectraMax M2e plate reader (Molecular Devices). Linear regression was used to extrapolate the amount of inhibitor required to abrogate enzyme activity. The association rate constant of inhibitor with enzyme (*k*_ass_) was measured by reaction progress curves under pseudo-first-order conditions for 4 h at 25°C with a final concentration of 50–250 nM inhibitor, 1 mM substrate and 5 nM bovine trypsin.

### α_1_-AT–trypsin complex stability assays

First-order dissociation rates were determined by continuously monitoring protease dissociation [55] using a chromogenic reporter substrate. α_1_-AT–trypsin complexes were formed by incubating 0.5 μM α_1_-AT with 0.5 μM bovine trypsin in assay buffer [20 mM Tris, 100 mM NaCl, 0.1% (w/v) PEG 8000, 10 mM CaCl_2_, pH 8.0] at room temperature for 1 h. Complexes were then diluted in the same assay buffer containing 1 mM *N*α-benzoyl-l-arginine 4-nitroanilide hydrochloride substrate (Sigma-Aldrich), to a final volume of 200 μl. Complex dissociation was monitored by following the increase in absorbance at 405 nm using a SpectraMax M2e plate reader (Molecular Devices) at 25°C for 4 h. Proteinase-only controls were included to convert the rate of change in absorbance to active proteinase concentration.

### Peptide incorporation experiments

It has previously been noted that a model peptide corresponding with the RCL of anti-thrombin, when incorporated into α_1_-AT, causes an increase in intrinsic protein fluorescence [41]. Wild-type α_1_-AT at 0.5 mg ml^−1^ in PBS was correspondingly incubated with a 50-fold molar concentration of an 11-mer (Ac-SEAAASTAVVI-NH2) and 4-mer (Ac-FLAA-NH2) peptide, at 37°C, in a 50 µl volume. Peptide incorporation was monitored for 24–48 h with excitation at 290 nm and emission at 330 nm, on a SpectraMax Gemini plate reader (Molecular Devices) using a half-well plate with a UV-transparent base (µClear, Greiner). Samples were degassed prior to the experiment and overlaid with VaporLock (Qiagen). Peptide incorporation was confirmed at the end of the experiment by non-denaturing PAGE.

### Snap refolding experiments

M and Z α_1_-AT were denatured for a minimum of 4 h at room temperature at a concentration of 10 mg ml^−1^ in 6 M guanidine hydrochloride. Aliquots of 20 µl were placed in the lid of a microcentrifuge tube containing 1 ml of Tris-buffered saline (TBS) with or without 0.6 mg ml^−1^ mAb_4B12_. Refolding was initiated by a rapid flip of the tube followed by several gentle inversions. Aliquots of 2.5 µl were removed at various time points between 0 and 240 min, diluted with 60 µl of TBS, and a 1.25-fold molar excess of trypsin. After a 5 min incubation, Suc-AAPV-pNa substrate at a final concentration of 400 µM was added and the rate of increase in absorbance at 405 nm was recorded. For each refolded sample, the percentage of inhibitory activity was calculated with reference to an identical non-denatured sample from the rates of substrate turnover (*V*):

$$\mathrm{Activity}=100\times (V_{\mathrm{protease}\;\mathrm{alone}}-V_{\mathrm{sample},\mathrm{refolded}})/(V_{\mathrm{protease}\;\mathrm{alone}}-V_{\mathrm{sample},\;\mathrm{non}-\mathrm{refolded}})$$

## Results

### The activity of mAb_4B12_ is not a consequence of conformational selectivity

**Figure 1.**
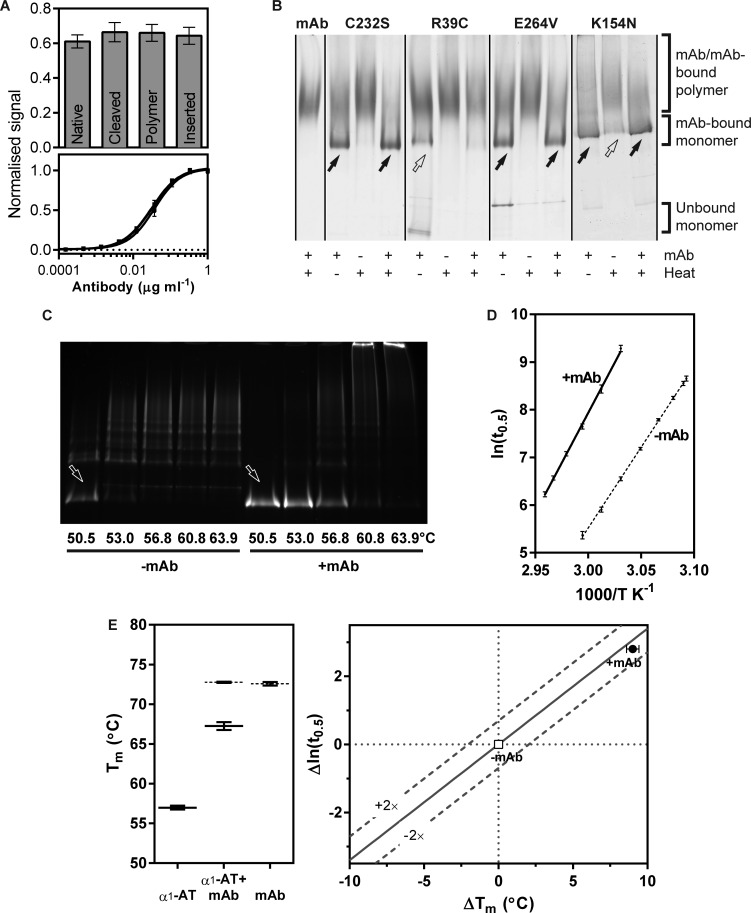
Effect of mAb_4B12_ on α_1_-AT polymerisation and stability. (**A**) A sandwich ELISA with rabbit anti-α_1_-AT polyclonal as the capture antibody, conformational variants of α_1_-AT as antigens and mAb_4B12_ as the detection antibody. Top: Absorbance at 50 µg ml^−1^ mAb_4B12_ is shown, normalised to the plateau value at maximal binding (SD, *n* = 2). Bottom: The normalised binding curves for the different conformers. (**B**) Variants of α_1_-AT (0.1 mg ml^−1^) were allowed to form a complex with a 2-fold molar excess of mAb_4B12_ for 15 min before (‘mAb+’) or after (‘mAb−’) heating at 55°C for 4 h (‘heat’), and resolved by non-denaturing PAGE, which separates both according to size and intrinsic charge. Antibody binding induces a cathodal (upwards) mobility shift. Polymerised recombinant α_1_-AT is well known to form poorly migrating, indistinct higher-order species [85]; thus, in this gel, a reduction in oligomerisation is most easily visualised as retention of the ‘mAb-bound monomer’ band (denoted by black arrows; outline arrows indicate a partial loss of intensity). Protein was visualised using Coomassie stain. Supplementary Figure S1A indicates the sites of these mutations. (**C**) α_1_-AT labelled at Cys232 with Alexa-488 and Alexa-594 was incubated at a concentration of 0.1 mg ml^−1^ over a range of temperatures for 12 h, in the absence and presence of a 2-fold molar concentration of mAb_4B12_. To ensure a consistent cathodal gel shift for ease of comparison, mAb_4B12_ was added to tubes from which it was absent during the incubation. Samples were resolved by non-denaturing PAGE, with labelled protein visualised by fluorescence under UV. Arrows indicate the position of antibody-bound monomer. (**D**) A 1:1 mixture of α_1_-AT labelled at Cys232 with Alexa-488 and Alexa-594 dyes was incubated at a concentration of 0.1 mg ml^−1^ at different temperatures in the absence and presence of a 2-fold molar concentration of mAb_4B12_. Polymerisation was monitored by the increase in FRET efficiency (representative curves are shown in Supplementary Figure S2C). The natural logarithm of the half-times of polymerisation was plotted against inverse absolute temperature, with SEM represented by error bars (*n* = 4). (**E**) Left: α_1_-AT_C232S_ in the presence and absence of a 1.5-fold molar excess of mAb_4B12_ was heated from 25 to 95°C at a rate of 1°C min^−1^, with the increase in fluorescence of SYPRO Orange reporting the transition to a polymerisation-prone intermediate [39]. Deconvolution of the resulting curves (Supplementary Figure S2D) provided values for the midpoint of the transition for α_1_-AT and mAb components (SEM, *n* = 6). Right: A stability–polymerisation graph [39] is shown, in which the change in thermal stability of α_1_-AT when bound to mAb_4B12_ is represented on the abscissa, and the relative change in polymerisation half-time is shown on the ordinate. Dashed lines represent the fold difference from the expected rate of polymerisation for a given change in thermal stability [SEM, *n* = 4 for Δln(*t*_0.5_) and *n* = 6 for Δ*T*_m_].

Our recent work described the 4B12 monoclonal antibody as a suppressor of α_1_-AT polymerisation. This involved the use of the monomeric form of Z α_1_-AT as the antigen to generate a hybridoma library, and its use in a primary screen that accordingly selected antibodies able to recognise this form of the protein [44]. A polymerisation-enhancing antibody, identified using a similar protocol, was found to exhibit marked conformational selectivity [22]. It is therefore possible that mAb_4B12_ too favours one conformation over others. To test this, the affinity of mAb_4B12_ for native, cleaved, polymeric and 11-mer RCL peptide-inserted forms of α_1_-AT was determined by ELISA and found to be indistinguishable (Figure 1A). Thus, mAb_4B12_ recognises an epitope that is present on — and is essentially unvaried between — native, loop-inserted and polymeric forms of α_1_-AT.

### mAb_4b12_ has activity against several α_1_-AT mutants

The Z (Glu342Lys) mutation is situated in a region termed the ‘breach’: the initial site of interaction between the inserting RCL and β-sheet A during the inhibition of a target protease. However, mutations that increase the tendency to polymerise are also found elsewhere in α_1_-AT. To determine whether mAb_4B12_ activity extends beyond the breach region, the antibody was assessed for its ability to prevent heat-induced polymerisation of the mild deficiency variants I (Arg39Cys), S (Glu264Val) and Queen's (Lys154Asn). These mutations localise to helix A, helix G and helix F, respectively (Supplementary Figure S1, top). An endpoint experiment was performed, in which variants were either preincubated with a 2-fold molar excess of mAb_4B12_ and then heated at 55°C for 4 h, or heated at 55°C prior to mAb_4B12_ addition. The well-characterised Cys232Ser mutant [39,46–49] (α_1_-AT_C232S_) was used as the control, and the result was evaluated by non-denaturing PAGE, in which polymerised α_1_-AT and mAb_4B12_-bound α_1_-AT appear higher on the gel (Figure 1B).

Without preincubation with the antibody, there was a loss of the monomer form upon heating for all variants. In contrast, mAb_4B12_ inhibited the formation of a higher-order polymeric population for α_1_-AT_C232S_, and the S and Queen's variants (Figure 1B). This effect was not evident for the I variant, despite possessing similar characteristics to S α_1_-AT [56]. The appearance of a band corresponding with a non-antibody-bound I variant suggests that this mutant interfered with complex formation. While the use of fixed experimental conditions does not exclude the possibility of further differences between mutants, it could nevertheless be concluded from these data that mAb_4B12_ can mitigate the consequences of mutations outside of the breach region.

### mAb_4b12_ has protease-dependent effects on inhibitory activity

mAb_4B12_ was previously found to reduce — but not to eliminate — inhibitory activity against human neutrophil elastase. This suggested that its binding site was situated in a region of the molecule only indirectly associated with the inhibitory mechanism [44]. In comparison, antibodies interacting with β-sheet A of protease nexin-1 were found to almost completely abolish inhibitory activity through direct interference with the RCL insertion mechanism [37]. Variable effects on inhibitory activity have been seen in studies with serpin mutants, dependent on the identity of the target protease [45,57–59]. To investigate whether this applied to mAb_4B12_ binding, the effect on the inhibition of trypsin by α_1_-AT was assessed. In contrast with elastase, the efficiency of the interaction, as represented by the SI, was almost identical in the presence and absence of antibody (1.17 ± 0.02 and 1.06 ± 0.03, respectively; SE of the linear regression, *n* = 4; Supplementary Figure S2A). Such contrasting behaviour has been shown in other studies to result from changes in the rate of RCL insertion during inhibition [58] or in the rate of deacylation due to variation in the interaction between a protease, its RCL tether and the region of the serpin in which it comes to rest [4,45,59]. Trypsin was subsequently selected as a tool with which to evaluate the effect of mAb_4B12_ on resistance to heat-induced inactivation.

### mAb_4b12_ maintains α_1_-AT in an active conformation

The ability of mAb_4B12_ to preserve α_1_-AT in a monomeric form, observed in Figure 1B, was confirmed by an experiment in which fluorescently labelled α_1_-AT_WT_ was incubated for 12 h over a range of temperatures. Analysis by non-denaturing PAGE revealed several temperatures that had induced marked sequestration into higher-order polymers in its absence, but not in its presence (Figure 1C). However, antibodies have been found that induce another serpin, PAI-1, to adopt an inactive yet monomeric latent conformation [33,34]. In the case of α_1_-AT, the latent conformation is a by-product of the polymerisation pathway that can be induced by heating [60] and augmented by the presence of buffer components that disfavour intermolecular interactions [61]. If the antibody was exerting an analogous effect — permitting conformational change while sterically preventing the association of single subunits — a comparable loss of activity might be expected to occur. α_1_-AT_WT_ was heated for 16 h at 55°C, and residual activity was determined by titration with trypsin. There was a ∼10% loss of inhibitory activity in the presence of mAb_4B12_ with respect to an unheated control, and a 75% decrease in its absence (Table 1 and Supplementary Figure S2B). Thus, binding substantially maintains α_1_-AT in the native state, rather than permitting the adoption of a monomeric yet inactive form.

**Table 1:** mAb_4B12_-mediated resistance to heat-induced inactivation The percentage of residual inhibitory activity of α_1_-AT against bovine trypsin was determined from the stoichiometry of inhibition after incubation for 16 h at 21 or 55°C, with a 2-fold molar excess of antibody added pre- or post-incubation. Errors denote SEM (*n* = 3).

Sample	Antibody added	21°C (%)	55°C (%)
α_1_-AT	Post-incubation	100	26.0 ± 7.2
α_1_-AT	Preincubation	89.3 ± 4.1	72.7 ± 0.0
mAb_4B12_	Preincubation	3.4 ± 1.7	6.8 ± 2.5

### Suppression of polymerisation occurs over a range of temperatures *in vitro*

mAb_4B12_ was found previously to be an effective inhibitor of polymerisation *in vitro* at 45°C and in mammalian cells at 37°C [44]. α_1_-AT labelled with Alexa Fluor-488 and Alexa Fluor-594 fluorophores can be used as a tool to follow polymerisation as a relative increase in FRET efficiency [39]. α_1_-AT_C232S_ (at 0.1 mg ml^−1^) was heated at temperatures between 50 and 64°C with, or without, a 2-fold molar concentration of mAb_4B12_. The resulting progress curves showed an antibody-mediated delay in the temperature at which polymerisation ensued (Supplementary Figure S2C). When the natural logarithm of half-times of polymerisation from the progress curves was plotted against the inverse absolute temperature, a linear relationship was apparent (Figure 1D). Interpolation at the mid-range temperature of 55°C revealed a decrease in the rate of polymerisation by 16.5-fold (±2.5 SEM, *n* = 4) due to mAb_4B12_ binding. The slopes of the lines permitted calculation of the apparent free energy barrier to polymer formation under the conditions of the experiment. At 349 ± 8 kJ mol^−1^ (SE of the linear regression, *n* = 4), this barrier was found to be modestly elevated in the presence of the antibody with respect to α_1_-AT_C232S_ alone (281 ± 4 kJ mol^−1^; SE of the linear regression, *n* = 4). As a result, assuming relative temperature insensitivity of these values within the supraphysiological temperature range considered, extrapolation of these relationships to 37°C gives a more pronounced (∼70-fold) decrease in the rate of polymerisation.

### mAb_4b12_ prevents conversion to a polymerisation-prone intermediate

Thermal stability experiments, in the presence of the environment-sensitive probe SYPRO Orange, report the transition from the α_1_-AT native state to a polymerisation-prone intermediate conformation along the polymerisation pathway [39]. Consequently, stabilisation of the native state will manifest as an increased midpoint of denaturation (*T*_m_), which in a two-protein system can be deconvoluted by fitting a double two-state unfolding equation [22] (Supplementary Figure S2D). α_1_-AT_C232S_ was incubated in the presence and absence of the antibody, with temperature increasing from 25 to 95°C at a rate of 1°C min^−1^. These experiments revealed a +10.3 ± 0.8°C (SEM, *n* = 6) enhancement in the stability of the mAb-bound α_1_-AT component (Figure 1E, left) against progression through the polymerisation-prone intermediate.

### The rate of polymerisation of antibody-bound α_1_-AT is consistent with effects on native stability

α_1_-AT shows a predictable decrease in the rate of heat-induced polymerisation with an increase in native-state stability [39]. Therefore, temperature can be used as a probe of mechanism: deviations from this trend indicate effects on other characteristics of the polymerisation pathway [22,39,62]. A comparison of polymerisation half-life interpolated at 55°C (Figure 1D) and stability determined by thermal unfolding (Figure 1E, left) is shown in Figure 1E (right). This analysis reveals a rate of polymer formation that is broadly consistent with that expected from the enhancement of native-state stability. Taking into account the presence of the epitope in both monomer and polymer, these collective data suggest that mAb_4B12_ exerts its effects primarily through changes in native-state stability. There is additionally a modest interference with the polymerisation mechanism, which is predicted to be more pronounced at lower temperatures.

### Residue Glu32 on helix A is proximal to the mAb_4B12_ epitope

**Figure 2.**
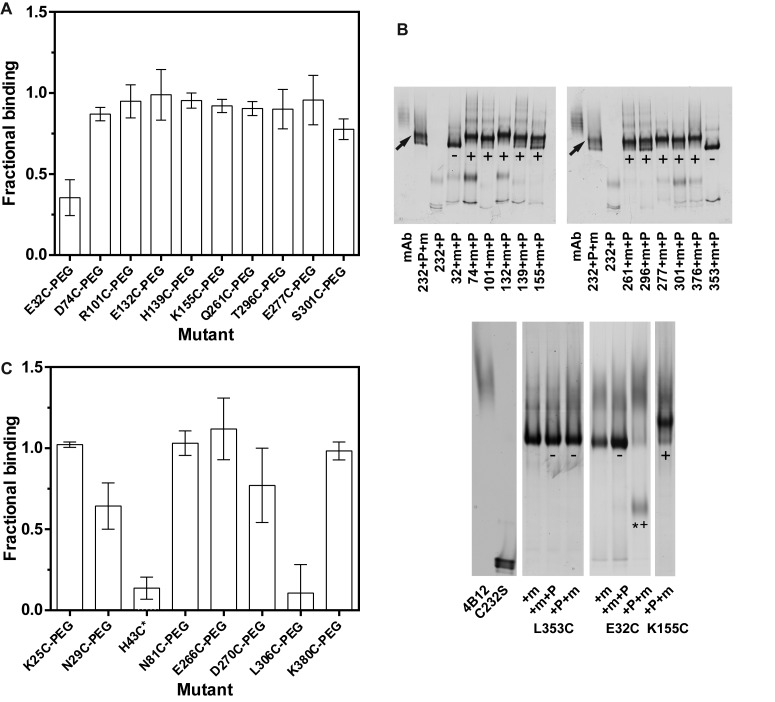
Epitope mapping by PEGylation. (**A**) Recombinant single-cysteine point mutants representing a sparse sampling of the α_1_-AT protein surface were generated and the effect of PEG5K conjugation on mAb_4B12_ binding was determined by a sandwich ELISA. Each binding curve was fit by a sigmoid function, from which the plateau absorbance was calculated; the fractional binding values are the ratio of the plateau absorbance in the absence and presence of the PEG5K moiety (SD, *n* = 3). (**B**) Top: Single-cysteine mutants, in complex with a 2-fold molar excess of antibody, were incubated with PEG5K maleimide and resolved by non-denaturing PAGE. Mobility shift due to conjugation (‘+’) or a lack thereof (‘−’) are indicated. Arrows indicate the position of an antibody-bound, pre-PEGylated control. Bottom: Mutants were incubated with a 2:1 ratio of mAb_4B12_ (+m) before (+m+P) or after (+P+m) PEGylation for 5 min and visualised by non-denaturing PAGE. A PEG-induced mobility shift (‘+’), a lack of PEG-induced shift (‘−’) and a lack of interaction with mAb_4B12_ (‘*’) are indicated. The K155C mutant is shown by way of reference and presented in full in Figure 5A. (**C**) A second, finer-grained screen of candidate binding positions by cysteine scanning and sandwich ELISA. The H43C value reported (‘*’) is the relative binding of the non-PEGylated mutant with respect to the non-PEGylated C232S control; this mutation of itself interfered with antibody complex formation. The ELISA results are summarised in Supplementary Figure S3.

The suppression of polymerisation in the context of an ability to bind both monomer and polymer suggests that mAb_4B12_ acts through modulation of a dynamic process. To glean further details of the underlying mechanism, we sought to locate the site of interaction. This initially involved a sparse screen of single-cysteine substitutions distributed around α_1_-AT. Ten candidate surface-exposed positions not involved in side chain-mediated interactions were selected (Figure 2A) with reference to the crystal structure of the native state [63]. Disruption of a putative binding site in the vicinity of each point mutation was then achieved by conjugation of a methoxypolyethylene glycol 5000 (PEG5K) maleimide moiety to the cysteine side chain. The ability of each PEGylated derivative to be bound by mAb_4B12_ was assessed by sandwich ELISA with respect to the non-PEGylated form. Due to the steric interference imposed by this bulky moiety, disruption manifested as a reduction in maximal binding, rather than a nuanced shift in affinity. This tranche of mutants identified α_1_-AT_E32C_-PEG5K as exhibiting reduced binding activity with respect to α_1_-AT_E32C_ (Figure 2A).

A thiol protection experiment was also performed, with each mutant pre-complexed to antibody followed by PEGylation. This was done to verify that the PEG5K group was not indirectly causing disruption of the epitope in these mutants. The complexes were then resolved by non-denaturing PAGE (Figure 2B, top). All mutants excluding α_1_-AT_L353C_ and α_1_-AT_E32C_ exhibited an upwards mobility shift characteristic of PEGylation. Parallel interference/protection experiments for these two mutants provided an inconclusive result for α_1_-AT_L353C_ due to the lack of PEGylation in all lanes (Figure 2B, bottom). In contrast, pre-PEGylation of α_1_-AT_E32C_ prevented a mobility shift diagnostic of mAb_4B12_ binding, and conversely pre-binding of antibody again blocked PEGylation of this variant. Notably, this position is in proximity to the Arg39Cys (I) mutation that appeared to exhibit reduced binding to mAb_4B12_ (Figure 1B) and lies within a region structurally conserved between native and loop-inserted conformations [44].

### The epitope spans helices A and I on the opposite face of α1-AT to β-sheet A

**Figure 3.**
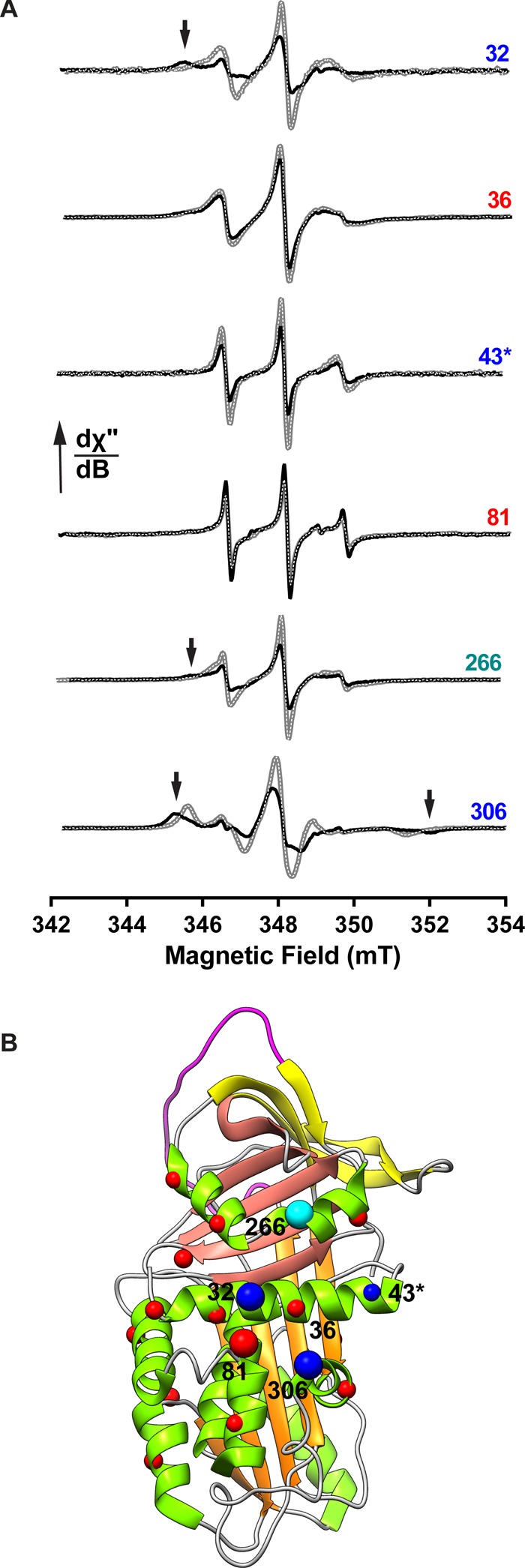
A survey of the mAb_4B12_ interface using CW-EPR. (**A**) CW-EPR spectra of 3-(2-iodoacetamido)-proxyl-labelled cysteine mutants were collected in the absence (grey with overlaid dashed line) or presence (black) of an equimolar amount of mAb_4B12_. First-derivative plots, normalised to their double integral, are shown. Arrows denote a substantive shift of the free radical probe to a low-mobility regime. (**B**) The combined results of the PEGylation and CW-EPR experiments are summarised, with the larger spheres indicating positions considered using both approaches. Those coloured red denote sites exhibiting little or no disruption of binding upon PEGylation or a lack of significant change in CW-EPR probe spectra. Blue spheres represent sites at which PEGylation disrupts antibody binding or that show a corresponding significant change in CW-EPR probe mobility. The cyan sphere indicates a site found from PEG experiments to be outside the binding site, yet also exhibiting a change in CW-EPR probe mobility. The asterisk indicates the site whose mutation disrupts antibody binding. Prepared using Chimera [86].

Based on this preliminary observation, a finer-grained cysteine scan was performed of nine residues in the vicinity of Glu32 — on helices A, C, G, H and I — with sites again selected based on the accessibility and lack of significant side chain contacts. Position 36 failed to conjugate with the PEG5K moiety and was excluded from the experiment. An ELISA-based assessment of the maximal binding of the variants by mAb_4B12_ demonstrated perturbation of the interaction upon PEGylation at 306 (Figure 2C). Strikingly, the *unconjugated* α_1_-AT_H43C_ mutant also demonstrated a lack of binding activity with respect to the α_1_-AT_C232S_ control. Overall, these results (summarised in Supplementary Figure S3 and Figure 3B) support the proximity of Glu32 and Leu306 to the epitope, and imply a critical role for the histidine side chain at position 43 in binding.

### Evaluation of the antibody-binding site by CW-EPR spectroscopy

The bulky PEG5K moiety had been chosen to increase the likelihood of identifying a binding site despite sparse sampling at the α_1_-AT surface. In contrast, site-directed spin labelling can be used as a sensitive and direct reporter of protein–protein interactions, with minimal perturbation of the interface due to small probe size [64]. Mutants corresponding with Glu32, Leu306 and His43, and two flanking sites from the PEGylation experiments, Glu266 and Asn81, were labelled with a 3-(2-iodoacetamido)-proxyl-free radical label, and CW-EPR spectra were collected in the presence and absence of mAb_4B12_ (Figure 3A). One of the key determinants of the morphology of a CW-EPR spectrum is spin probe mobility, which relates to spectral width. Here, interaction with the antibody resulted in the appearance of a distinct species, reflected by a marked increase in spectral broadness and reduction in intensity, for both α_1_-AT_E32C_ and α_1_-AT_L306C_. Additional effects were seen at position 266, at which site PEG5K conjugation had not prevented binding (Figure 2C), therefore likely delineating one edge of the interface. The other tested sites did not show significant peak broadening (Figure 3A). One surprising result is the lack of change reported by the label at residue 36, given its situation within the triangle delineated by 32-43-306. However, we note that an antibody–antigen interface is neither uniform nor completely planar; it may be that occlusion by the antibody does not significantly alter its behaviour. Overall, these results confirm the involvement of positions 32 and 306 at the α_1_-AT/mAb_4B12_ interface. The combined results from the PEGylation and CW-EPR experiments are summarised in Figure 3B.

### Properties of the antibody-binding interface evaluated by thermal shift experiments

The stark increase in thermal stability of antibody-bound α_1_-AT (Figure 1E, left) was used as a further tool to evaluate the effect of mutations on binding and the polymerisation suppression mechanism. First, the role of the His43 side chain in the interface was verified. In contrast with the α_1_-AT_C232S_ control, the α_1_-AT_H43C_ mutant failed to exhibit a comparable increase in *T*_m_ in the presence of mAb_4B12_, consistent with the lack of interaction between these components (Table 2 and Supplementary Figure S4A). To confirm that this was due to the loss of a histidine — rather than the acquisition of a cysteine side chain — an additional α_1_-AT_H43A_ mutant was generated. This variant displayed an identical behaviour with that of α_1_-AT_H43C_. Therefore, the histidine side chain at position 43 is an obligate component of the epitope.

**Table 2:** Thermal stability of α_1_-AT variants in the presence and absence of mAb_4B12_ Control and His43 variants were heated between 25 and 95°C with a temperature increment of 1°C min^−1^ in the presence of SYPRO Orange, with and without a 2-fold molar excess of mAb_4B12_. Deconvolution of the resulting fluorescence curves yielded values for the midpoint of the transition for α_1_-AT and mAb components. Errors reflect SD (*n* = 2–6).

Sample	α_1_-AT (°C)	mAb_4B12_ (°C)
α_1_-AT_C232S_	57.0 ± 0.6	–
α_1_-AT_C232S_ + mAb_4B12_	67.3 ± 1.2	72.8 ± 0.2
mAb_4B12_	–	72.6 ± 0.6
α_1_-AT_H43C_	57.6 ± 1.6	–
α_1_-AT_H43C_ + mAb_4B12_	58.4 ± 1.3	72.8 ± 0.3
α_1_-AT_H43A_	59.1 ± 0.4	–
α_1_-AT_H43A_ + mAb_4B12_	59.4 ± 0.3	73.2 ± 0.0

### Disulphide constraints at the termini of helix I do not replicate mAb_4B12_ behaviour

**Figure 4.**
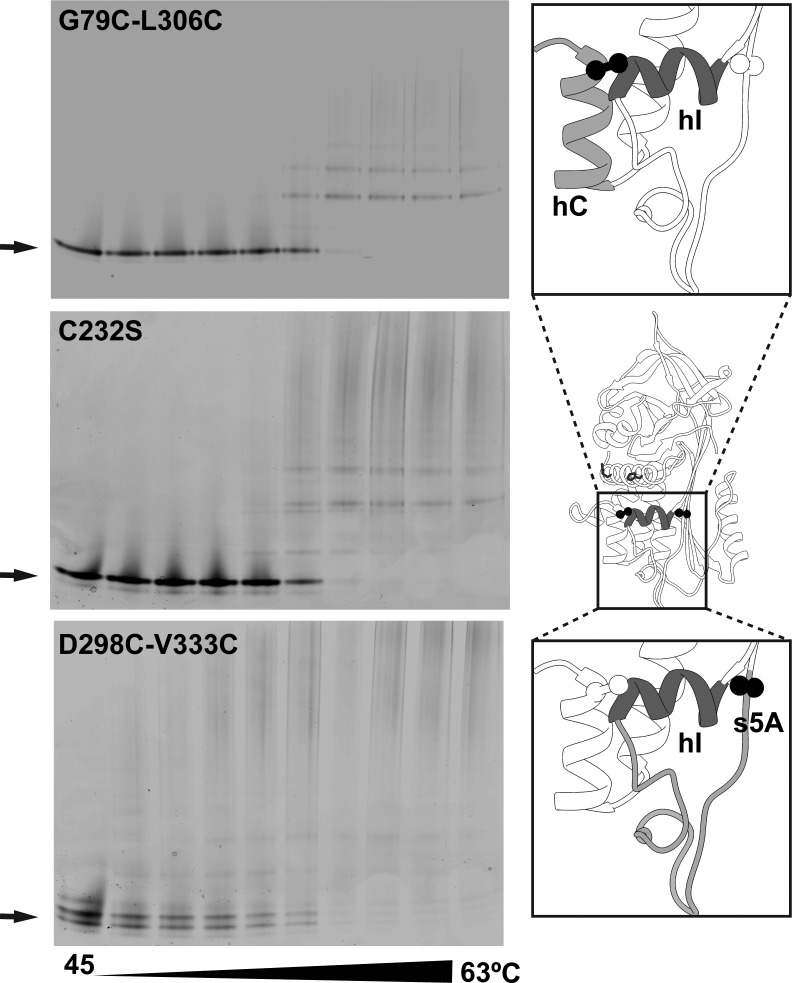
The effect of introduced disulphides on polymerisation. Non-denaturing PAGE was used to resolve the monomeric and oligomeric states following an experiment in which variants (0.1 mg ml^−1^ in PBS) were heated for 4 h at temperatures between 45 and 63°C. Polymerised recombinant α_1_-AT forms poorly migrating, indistinct higher-order species [85]; thus, oligomerisation is most easily observed through loss of monomer band (indicated by arrows). Protein was visualised using Coomassie stain. The positions of the introduced disulphides with respect to helix I (hI; dark grey) and adjacent structural elements (helix C and strand 5A; light grey) are shown in the panels on the right.

It has been observed that constraints imposed by introduced disulphides generally result in global thermal stabilisation of α_1_-AT with a consequent increase in resistance to polymerisation [39,65]. One possibility is that mAb_4B12_ binding could be mimicking this effect. Of the two structural elements within the epitope — helices A and I — the latter is considered the more labile element [27]. Indeed, it has been proposed to be denatured in a polymerisation-prone unfolding intermediate [66,67], and according to one model of polymerisation, it is expected to be extricated from the molecule and fully denatured in the antitrypsin polymer [29]. To test the possibility that the antibody merely acts by preventing displacement of helix I from its native position, two disulphides were engineered into two separate molecules: α_1_-AT_79-306_, tethering position 306 at the C-terminus of helix I to residue 79 on helix C; and α_1_-AT_298-333_ that tethers the N-terminus of helix I to β-strand 5A (Figure 4, right panels). In the resulting mutants, the disulphides were present in the majority (∼90 and ∼60%, respectively) of molecules, as determined by a difference in SDS–PAGE migration between reduced and non-reduced samples.

Thermal denaturation experiments were undertaken; despite the mixed population of α_1_-AT_298-333_, the thermal stability curve was well described by a single two-state unfolding equation. In contrast with typical observations with disulphides [39], α_1_-AT_79-306_ and α_1_-AT_298-333_ did not demonstrate an increase in global native stability with respect to the α_1_-AT_C232S_ control, instead exhibiting a reduction in *T*_m_ of −1.2 ± 0.6 and −3.5 ± 0.7°C, respectively (Table 3 and Supplementary Figure S4B; SEM, *n* = 5–6). Therefore, any difference observed in the polymerisation propensity would be ascribable to specific effects of a disulphide on the polymerisation pathway, rather than a global stabilisation effect [39]. This was investigated by performing endpoint polymerisation experiments [49], with monomeric protein heated for 4 h at temperatures ranging between 45 and 63°C, and samples were analysed by non-denaturing PAGE. The resulting profiles were found to be almost the same for the control α_1_-AT_C232S_ protein and the disulphide mutants (Figure 4, left panels). It was noted that there was no persistence of a fraction of the monomer for α_1_-AT_298-333_ that might be attributable to the disulphide-bonded population. These data indicate that constraining helix I at either terminus does not stabilise the native conformation against polymerisation, strongly suggesting that this is not the basis for the activity of mAb_4B12_.

**Table 3:** Thermal stability of α_1_-AT disulphide variants in the presence and absence of mAb_4B12_ Disulphide variants were heated between 25 and 95°C at a rate of 1°C min^−1^ with and without a 2-fold molar excess of mAb_4B12_, and unfolding monitored using SYPRO Orange fluorescence. The resulting fluorescence curves yielded values for the midpoint of the transition for α_1_-AT and mAb components (*T*_m_). ‘DTT’ denotes samples reduced using 40 mM DTT for 15 min prior to dilution (with a final DTT concentration <1 mM) and complex formation with mAb_4B12_. Errors reflect SEM (*n* = 3–6).

Sample	−DTT		+DTT	
	α_1_-AT (°C)	mAb_4B12_ (°C)	α_1_-AT (°C)	mAb_4B12_ (°C)
α_1_-AT_79-306_	55.8 ± 0.9	–	54.0 ± 0.3	–
α_1_-AT_79-306_ + mAb	61.4 ± 0.6	72.5 ± 0.3	63.3 ± 1.0	71.7 ± 0.3
α_1_-AT_298-333_	53.4 ± 1.0	–	52.4 ± 0.6	–
α_1_-AT_298-333_ + mAb	62.8 ± 0.9	72.6 ± 0.4	67.2 ± 1.7	72.8 ± 0.8
α_1_-AT_191-339_	61.3 ± 0.5	–	56.9 ± 0.4	–
α_1_-AT_191-339_ + mAb	71.2 ± 0.7	72.6 ± 0.2	68.5 ± 1.2	72.1 ± 0.5

### Antibody-mediated stabilisation is suppressed by the presence of disulphide constraints

The effect of introduced disulphides on the ability of mAb_4B12_ to confer enhanced thermal stability was assessed. An additional mutant that prevents sheet opening at the top of β-sheet A, α_1_-AT_191-339_ [39], was also considered. This (>90% disulphide-bonded) mutant exhibited an increase in *T*_m_ to 71.2 ± 0.7°C (SD, *n* = 5) when bound to mAb_4B12_ (Table 3 and Supplementary Figure S4B). In contrast, the presence of the 79–306 and 298–333 disulphides yielded a *T*_m_ increase for the mAb_4B12_ complex attenuated by −6.9 and −4.5°C with respect to the antibody-bound α_1_-AT_C232S_ control. This tentatively suggested that these constraining disulphides antagonised the ability of mAb_4B12_ to stabilise the native state. In support of this, treatment with DTT did indeed cause α_1_-AT_298-333_ + mAb_4B12_ to manifest increased stability to within −0.1°C of the α_1_-AT_C232S_ + mAb_4B12_ control.

In contrast, reduction with DTT led to a milder +1.9°C increase in the *T*_m_ of α_1_-AT_79-306_ + mAb_4B12_, such that it remained −4.4°C lower than the control and the reduced α_1_-AT_298-333_ + mAb_4B12_ sample. This suggested that the Gly79Cys/Leu306Cys double mutation itself contributed substantially to the limited mAb_4B12_ stabilisation. As 306 is situated within the epitope, it is possible that this variant is bound with less affinity by the antibody. To check this, an ELISA was performed (Supplementary Figure S4C). Under the conditions of the ELISA, comparable EC_50_ values were found for α_1_-AT_C232S_ (10.8 ± 0.4 ng ml^−1^), α_1_-AT_298-333_ (9.1 ± 0.7 ng ml^−1^) and α_1_-AT_191-339_ (12.2 ± 0.9 ng ml^−1^), making these variants directly comparable. In contrast, while the antibody did recognise α_1_-AT_79-306_, there was a modest ∼1.6-fold increase in the EC_50_ of the interaction to 17.1 ± 1.8 ng ml^−1^ (±SEM of the regression, *n* = 3). Despite rendering interpretation of the effect of α_1_-AT_79-306_ on mAb_4B12_-induced stabilisation non-trivial, this result highlights the contribution of residues in this region to the mAb_4B12_ epitope and is consistent with the limited recovery of *T*_m_ upon reduction in the 79–306 disulphide.

Taken together, the results in Table 3 and Figure 4 indicate that a reduction in the mobility of the helix I termini does not adequately account for the observed resistance to polymerisation. Rather, the mechanism of stabilisation by mAb_4B12_ is antagonised by a disulphide constraint at the helix I N-terminus, but not by one in the breach region at the top of β-sheet A.

### Propagation of structural change to β-sheet A

**Figure 5.**
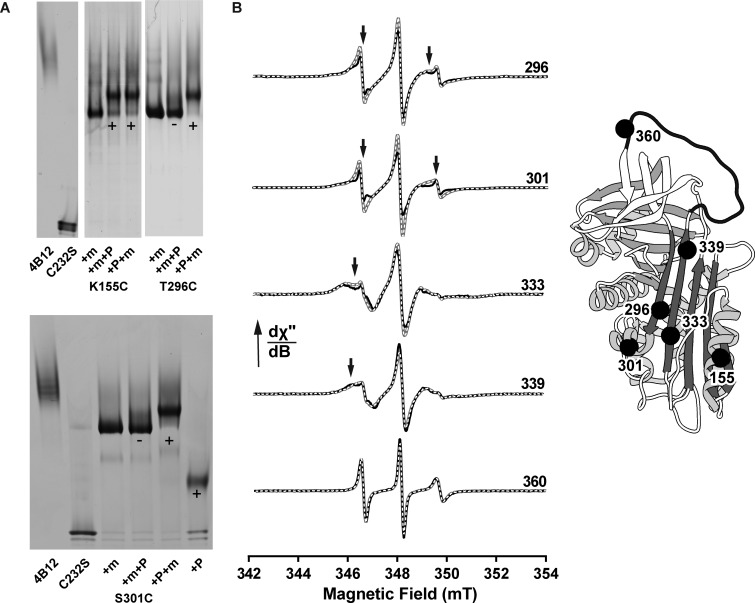
mAb_4B12_ binding induces changes in α_1_-AT beyond the epitope. (**A**) Single-cysteine mutants on helix I (S301C), at the strand 6A-helix I interface (T296C) and on helix F (K155C) were incubated with a 2:1 ratio of mAb (+m) before (+m+P) or after (+P+m) PEGylation for 5 min and visualised by non-denaturing PAGE. Those exhibiting a PEG-induced mobility shift (+) or lacking a shift (−) are indicated. (**B**) CW-EPR spectra of 3-(2-iodoacetamido)-proxyl-labelled cysteine mutants on the RCL–β-sheet A axis (T296C, S301C, K333C, T339C and I360C — shown in the panel on the right) in the absence (grey with overlaid dashed line) and presence (black) of an equimolar concentration of mAb_4B12_. The experiment was performed as in Figure 3. A change in probe behaviour is indicated by an arrow.

During sparse screening to delineate the epitope, some α_1_-AT cysteine mutants yielded intermediate results in the protection assay when compared with α_1_-AT_E32C_ (Figure 2B, top). Subsequent parallel binding interference/protection assays (as performed in Figure 2B, bottom) identified two sites, position 301 on helix I, and residue 296 at the junction between helix I and strand 6A, that exhibited a distinct behaviour (Figure 5A). These positions could be fully PEGylated in the absence of mAb_4B12_, and the PEG5K group did not prevent binding of mAb_4B12_, but when the antibody was bound first, these mutants exhibited a reduced ability to accept the PEG5K maleimide. This reduction in susceptibility to PEG5K conjugation was not intrinsic to the presence of antibody, as cysteines introduced at different locations (including residue 155, on helix F) showed a full mobility shift (Figure 5A).

In light of the observations with a tethered helix I N-terminus, the previously noted mAb_4B12_ induced reduction in inhibitory activity against neutrophil elastase [44] and the effect of helix I mutations on inhibitory activity [67], these derivatisation results raise the possibility that antibody binding mediates β-sheet A behaviour through structural changes in this region. To assess this possibility directly, spin labels were used as sensitive reporters of differences (primarily mobility and polarity) in the local environment. In addition to 296 and 301, three other positions along the RCL-β-sheet A axis were chosen (Figure 5B). Upon antibody complex formation, for α_1_-AT_T296C_, α_1_-AT_S301C_ and α_1_-AT_V333C_, there was a change reported in the environment of the spin probe, resulting in the adoption of an altered mobility regime. The effect was more marginal for α_1_-AT_T339C_, consistent with the lack of interference of α_1_-AT_191-339_ with antibody-mediated stabilisation, and was absent for α_1_-AT_I360C_. These results collectively suggest propagation of conformational change to the lower region of β-sheet A.

### β-sheet A becomes ‘permissive’ upon antibody binding

**Figure 6.**
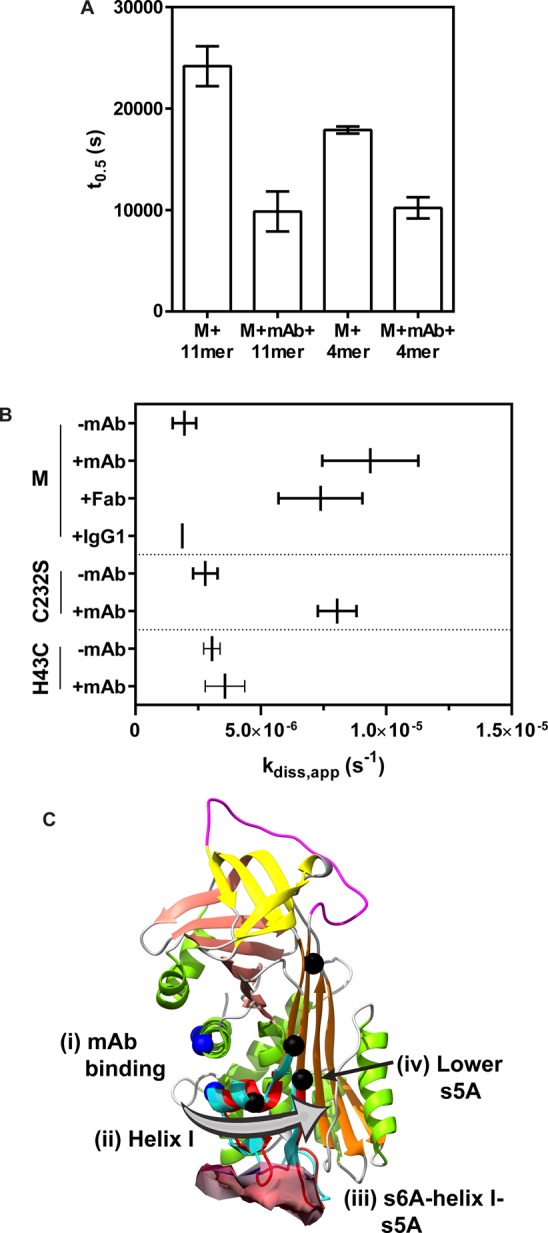
Induced changes in β-sheet A behaviour. (**A**) The incorporation of a 50-fold molar excess of synthetic 11-mer (Ac-SEAAASTAVVI-NH2) and 4-mer (Ac-FLAA-NH2) peptide by 0.5 mg ml^−1^ α_1_-AT was monitored by a change in intrinsic protein fluorescence [41] at 37°C. Half-times of fluorescence change (*t*_0.5_) were calculated using a single exponential equation (SEM, *n* = 4–6) from time-course data such as that shown in Supplementary Figure S5A. (**B**) Complexes were formed between α_1_-AT variants and bovine trypsin, and the regain of protease activity was monitored following dilution from 0.5 µM to between 1.6 and 50 nM. Representative time-course data are shown in Supplementary Figure S5B. The rate of dissociation was calculated (SD, *n* = 3) for plasma-derived wild-type (M), α_1_-AT_C232S_ control and α_1_-AT_H43C_ in the presence and absence of mAb_4B12_, the corresponding Fab fragment and an IgG1 isotype control. (**C**) The proposed mechanism of action of mAb_4B12_, where (i) binding (ii) induces a change in helix I which (iii) propagates to β-sheet A via strand 6A and/or the loop connecting helix I with strand 5A, (iv) shifting the lower region towards a more open ‘permissive’ conformation. It is proposed that this occurs out-of-sequence with other changes necessary for polymerisation, and consequently, α_1_-AT adopts a partially stabilised character. Residues of the epitope are shown in blue, those that report indirect change upon binding are black, the site of the interface between serpin and enzyme in the inhibitory complex is shown as a partial surface and the shift that occurs in the lower portion of β-sheet A during RCL insertion is highlighted by a cyan ribbon.

The ability of peptide mimetics of the RCL to antagonise polymerisation represented key early evidence of the involvement of the RCL in polymer formation [6,31]. While a peptide derived from one serpin can anneal with another, different rates of incorporation have been observed between the M and Z α_1_-AT variants, interpreted to be the result of altered β-sheet A dynamics [41]. To explore the possibility that mAb_4B12_ binding precipitated a comparable behaviour in this region, the rate of incorporation of two model peptides, an 11-mer and 4-mer, was determined from progress curves reporting intrinsic tryptophan fluorescence [41,68] (Supplementary Figure S5A). These data were satisfactorily fit by a single-phase exponential equation, and surprisingly showed a 2.5- and 1.8-fold increase in the rate of peptide incorporation in the presence of antibody with respect to the non-antibody-bound control (Figure 6A). Thus, contrary to expectations, antibody binding appears to increase β-sheet A liability.

### mAb_4b12_ causes changes in trypsin–α_1_-AT complex stability

An altered rate of association between a serpin and a protease during inhibition can provide a measure of changes in RCL/β-sheet A behaviour. This is exemplified by anti-thrombin, whose partially inserted RCL is expelled upon interaction with heparin, altering its inhibitory kinetics towards proteases in the coagulation cascade [69,70]. Similarly, it has been proposed that the Z variant of α_1_-AT may also exhibit partial insertion in association with its polymerisation-prone nature [41], a change also predicated by the C-terminal polymerisation mechanism [30].

To understand whether such effects are seen in response to antibody binding, progress curves for the inhibition of trypsin under pseudo-first-order conditions were recorded. However, these showed an atypical morphology, suggestive of a significant degree of serpin–enzyme complex dissociation [55]. To explore this possibility further, complex stability experiments were performed: α_1_-AT and trypsin were combined at a high concentration, and rapidly diluted to low concentrations in the presence and absence of antibody, with regain of enzyme activity followed as a function of time (Supplementary Figure S5B). The recovery of protease activity was indeed found to be stimulated in the presence of mAb_4B12_, with a dissociation rate (*k*_diss_) increased by 4.8-fold and 2.9-fold for plasma and recombinant α_1_-AT, respectively (Table 4; indicated by a shift to the right in Figure 6B). This was not observed in the absence of antibody binding, using an IgG1 isotype control, or for the binding-compromised α_1_-AT_H43C_ mutant. In addition, the Fab fragment derived from mAb_4B12_ showed a comparable effect on complex dissociation, rendering it unlikely that the antibody bulk was exerting a steric influence on the final resting position of the protease. The minimal effect on SI with trypsin indicates that the progression from encounter to inhibited complex occurs with sufficient efficiency; therefore, accelerated complex dissociation is consistent with perturbation at the base of β-sheet A in the region where the inhibited protease comes to rest.

**Table 4:** The rate of α_1_-AT–trypsin complex dissociation Complexes between α_1_-AT variants and bovine trypsin at an initial concentration of 0.5 µM were diluted to between 1.6 and 50 nM and the regain of protease activity monitored using a chromogenic substrate. The first-order rates of dissociation are shown in the presence and absence of mAb_4B12_, its Fab domain and an isotype control (SD, *n* = 3).

Sample	−mAb_4B12_ × 10^−6^ s^−1^	+mAb_4B12_ × 10^−6^ s^−1^	+Fab_4B12_ × 10^−6^ s^−1^	+IgG1 × 10^−6^ s^−1^
α_1_-AT_WT_	2.0 ± 0.5	9.4 ± 2.0	7.4 ± 1.7	1.9 ± 0.2
α_1_-AT_C232S_	2.8 ± 0.5	8.0 ± 0.8	–	–
α_1_-AT_H43C_	3.1 ± 0.3	3.6 ± 0.8	–	–

### mAb_4b12_ assists the refolding of the Z variant

Examples exist of monoclonal antibodies that assist in folding of an antigen to a native, active conformation [71], and it is possible that this is the basis of the activity of the single-chain 4B12 analogue in cells [44]. To test this possibility, wild-type (M) and Z α_1_-AT were denatured at 10 mg ml^−1^ in 6 M guanidine and snap-refolded with a 50-fold dilution in the presence and absence of a 2-fold concentration of mAb_4B12_. The inhibitory activity at various time points was determined by incubating for 5 min with a 1.25-fold molar excess of bovine trypsin. For each sample, residual activity was calculated as a proportion of that of an identical but non-denatured control. While it was not possible to derive accurate refolding rates from these data, M and Z variants with mAb_4B12_, and M without, showed a trend of increasing inhibitory activity over time; in contrast Z alone did not (Supplementary Figure S5C). Correspondingly, the resulting steady-state levels of activity for the M samples (−mAb_4B12_: 55 ± 2%, +mAb_4B12_: 55 ± 4%; SEM of the fit, *n* = 3) and Z + mAb_4B12_ (60 ± 5%) were similar, and higher than that of Z-mAb_4B12_ (42 ± 1%; Supplementary Figure S5D).

## Discussion

Mutations in the SERPINA1 gene result in attenuated secretion of α_1_-AT by hepatocytes, due to the formation of polymers in the ER [72] and proteasomal degradation of misfolded protein [32]. These polymers have been shown to consist of hyperstable, folded protein in an unbranched arrangement [73] and form through self-association of a polymerisation-prone monomeric species [21,22]. However, there is debate as to whether polymers arise from a near-native conformation *in vivo* or whether the polymerisation-prone species is a substantially unfolded intermediate on the folding pathway [29,30,44,60]. In this regard, it is notable that a single-chain variant of a monoclonal antibody that prevents polymerisation from native material *in vitro* is also effective during co-expression of a polymerisation-prone variant of α_1_-AT in a cell model of disease [44]. Here, we have sought to characterise this antibody further to understand its mechanism of action, and the implications for the polymerisation pathway that it antagonises.

Despite the use of monomeric Z α_1_-AT in the process that identified mAb_4B12_, this antibody was found to suppress polymerisation for deficiency variants with mutations at different locations within the molecule (Figure 1B). Based on evidence that these variants assert their effects on the molecule via a common mechanism [26,56], this indicates that the action of the antibody is mechanism- (rather than variant-) centric. By ELISA, mAb_4B12_ was demonstrated to lack conformational selectivity (Figure 1A), precluding action as a conformational ‘sink’ that would stabilise, for example, the native state over others. This is suggestive of a site of binding which is relatively invariant between the 5-stranded native and 6-stranded inserted conformations of the protein, and correspondingly that it is modulation of dynamic changes that form the likely basis of its mode of action. Consistent with this, the epitope, identified by indirect and direct measures of binding (Figures 2 and 3), was found to span helices A and I. These elements are present in a region of the protein that shows high similarity between crystal structures of the native and cleaved conformations [44]. Helix I itself, however, has been proposed to undergo plastic deformation during insertion of a cleaved RCL [74], and mutations within it have been shown to affect inhibitory activity [67]. It is notable then that mAb_4B12_ activity was antagonised upon tethering of this element by an introduced disulphide linking positions 298 and 333 (Table 3). In addition, antibody binding was associated with a procession of indirect conformational effects along the helix I–β-sheet A axis (Figure 5A,B), with a concomitant increase in thermal transition temperature of ∼10°C (Figure 1E, left). These effects manifested an altered behaviour in β-sheet A, reflected by a protease-dependent perturbation of inhibitory efficiency [44], increased ability to accommodate exogenous RCL mimetic peptides (Figure 6A), perturbation of the α_1_-AT–trypsin complex (Figure 6B) and altered strand 5A dynamics (Figure 5B).

It is noteworthy that the mAb_4B12_ epitope is adjacent to the binding site of a monoclonal antibody that stabilises the serpin PAI-1 against inactivation, which includes the helix I-strand 5A loop [36], and one that confers substrate-like behaviour through direct interference with a covalently bound protease [35]. Antibodies that decrease PAI-1 stability have also been found that interact with regions associated with conformational transitions: the C-sheet upon strand 1C displacement [33] and helix D [34]. Others that suppress inhibitory activity have been noted to interact directly with components of the inhibitory apparatus, localising to the vicinity of helix F of PAI-1 — which is known to move during RCL insertion [38] — and the lower portion of β-sheet A that accepts the incoming RCL during inhibition by protease nexin-1 [37]. Each of these epitopes is distinct from the one presented here, and collectively they span a significant proportion of the serpin fold.

Proposed models of polymerisation differ in the degree of structural perturbation they predict, such as unravelling of the C-terminus [30] or unfolding of helix I and strand 5A [29]. Release of strand 1C [39,46] and deformation of helix F [65,75,76] are additional changes that have been shown to occur during polymerisation. All models, however, anticipate adoption of an RCL-inserted state for β-sheet A, be it a consequence of intermolecular or intramolecular interactions (Figure 1A), and it is frequently expected or implied that the breach region [74] of β-sheet A plays a significant role [6,30,41,46,77]. For example, in anti-thrombin, heparin causes this sheet to close, expelling the partially inserted RCL from the ‘breach’ [69] and increasing the stability of the molecule by ∼7°C [78]. However, there does not appear to be significant involvement of the breach in the mAb_4B12_ mechanism (Table 3 and Figure 5B). Similarly, the binding of vitronectin by PAI-1 in the cleft that partitions helix E from helix F stabilises the molecule against inactivation by inducing a shift in β-sheet A towards the ‘closed’ state, evidenced by a reduced rate at which extrinsic peptide is incorporated [79]. This highlights a distinguishing feature of mAb_4B12_-mediated stabilisation: paradoxically, binding renders β-sheet A more receptive to peptide incorporation (Figure 6A).

Furthermore, along the sheet lies the shutter domain, involved in the regulation of its opening and identified from the clustering of mutations that compromise molecular activity or stability [10,73,80]. The site of influence of mAb_4B12_, in the distal portion of β-sheet A, flanks this region. It is proposed that mAb_4B12_ acts by inducing a shift to a ‘quasi-inserted’ conformation in the lower region of β-sheet A, near the shutter, with minimal disruption of the sheet in the breach region. The native α_1_-AT fold is peppered with unfavourable interactions whose resolution imbues the inserted state with a pronounced increase in thermodynamic stability [2]. Accordingly, it is proposed that the incomplete shift to an ‘open’ conformation, out-of-step with other changes that occur during polymerisation, results in a partial stabilisation of the protein by resolution of some of these unfavourable interactions (Figure 6C). Furthermore, it is suggested that this stabilisation is exerted primarily on the native state (Figure 1E), conferring enhanced kinetic stability; however, the proposed structural change would be expected to remain compatible with the polymerisation mechanism, due to the ability of the antibody to bind the polymer form.

Some observations presented permit further conclusions to be drawn with regard to the status of helix I in polymers induced *in vitro* and in cells. As shown here, mAb_4B12_ recognises polymer and monomer equally, with the C-terminus of helix I forming a component of its epitope (Figures 1A, 2 and 3), supported by the decreased affinity for a 79–306 double mutant (Supplementary Figure S4C). In addition, no stabilisation was observed against thermal transition or polymerisation when helix I was tethered using introduced disulphides (Figure 4). These data indicate that this structural element is likely to be substantially intact in the polymer. This is of particular relevance to the ‘β-hairpin model’, which extends implications from a crystal structure of an anti-thrombin dimer to α_1_-AT, based on limited proteolysis experiments and an introduced disulphide [29]. One of its core tenets is that helix I is substantially unwound. Therefore, while expectations of the relevance of this conformation to α_1_-AT have been curtailed [23,30,39], at the very least these data indicate that a re-appraisal of the model, in which an intact helix I is countenanced, is required.

It has not yet been established whether polymerisation predominantly occurs from a near-native state or during folding. Many lines of evidence favour the former: there is a well described absence of the UPR, indicating in turn an absence of an unfolded protein load [7,8,32]; expression in the absence of ER chaperones results in the appearance of atypical small circular oligomeric species [81]; there is a lack of the misfolded latent conformation in *ex vivo* tissue [60] and the molten globule-like unfolding intermediate [27] results in a polymer that is distinct immunologically [23], and most likely therefore structurally [25]. It is noteworthy that co-expression of a single-chain variant (scFv) of mAb_4B12_ results in a reduction in the intracellular polymerisation of Z α_1_-AT by 60% [44]. The ability of mAb_4B12_ to also suppress heat-induced polymerisation from a properly formed native conformation *in vitro* is suggestive of processes common to both environments. The discontinuous nature of the mAb_4B12_ epitope identified here provides evidence of a monomeric antigen that is substantially folded when it is bound. The observation that mAb_4B12_ can improve the yield of active Z α_1_-AT to a level comparable with the wild-type variant during refolding *in vitro* is supportive of a folding pathway that manifests its mutation-induced differences only once this structured, near-native state is achieved; therefore, this is the form that mAb_4B12_ most probably exerts its effects on *in vivo*.

The primary site of α_1_-AT polymer formation within the ER of hepatocytes renders antibody-based molecules unlikely therapeutic reagents for α_1_-AT deficiency. Nevertheless, use of mAb_4B12_ as a tool has identified a region that can allosterically regulate β-sheet A dynamics. Small molecules that assert allosteric effects have been approved for use in the clinic [82,83]; one of the key desirable properties they can exhibit is greater specificity than those targeted to highly conserved elements such as active sites [84]. The data presented here suggest a means — not directly involving the RCL or β-sheet A — by which polymerisation can be suppressed. Notably, while replication of an antibody–antigen interface is beyond the ability of small molecules, a relatively small component of the mAb_4B12_ epitope is associated with conformational liability. Taken together, the results presented here and our previous data obtained using a single-chain variant of mAb_4B12_ [44] suggest that polymerisation proceeds *in vivo* from a near-native conformation, and that the state of the molecule prior to the ‘polymer decision point’ is sufficiently well structured that it represents a tractable target for small-molecule intervention.

## Abbreviations

α_1_-AT, α_1_-antitrypsin; CW-EPR, continuous-wave electron paramagnetic resonance; ER, endoplasmic reticulum; FRET, Förster resonance energy transfer; *k*_ass_, second-order association rate constant; *k*_diss_, first-order dissociation rate constant; mAb, monoclonal antibody; PBS, phosphate-buffered saline; PEG5K, methoxypolyethylene glycol maleimide 5 kDa; RCL, reactive centre (or site) loop; scFv, single-chain antibody fragment; SI, stoichiometry of inhibition; TBS, Tris-buffered saline; *T*_m_, midpoint of thermal denaturation; UPR, unfolded protein response.

## Author Contribution

N.M.-S., J.A.I. and D.A.L. conceived the study. N.M.-S., J.A.I., A.M.J., E.S., C.W.M.K. and A.O. designed experiments. N.M.-S., J.A.I., A.M.J., M.L., S.V.F., A.S.N., J.L.W., E.S. and A.O. performed experiments. J.A.I., N.M.-S., D.A.L., A.M.J., M.L., S.V.F., A.S.N., E.S., J.L.W., C.W.M.K. and A.O. analysed/interpreted data. N.H.-C., I.H., E.M. and J.P. generated reagents. J.A.I., N.M.-S., A.M.J. and D.A.L. wrote the manuscript, which was edited and approved by all authors.

## Funding

N.M.-S. was supported by a Marie Curie IEF Fellowship. A.M.J. and S.V.F. are recipients of EPSRC/GlaxoSmithKline CASE Studentships. A.S.N. is a Pomona Scholar. E.M. is supported by the Sapienza University of Rome (Scientific Research calls 2014 and 2015). I.H. is an eALTA Fellow. This work was funded in part by a grant from the Alpha-1 Foundation to J.A.I. D.A.L. is funded by the Medical Research Council (UK) (G0901786) and the National Institute for Health Research/University College London Hospitals NHS Foundation Trust Biomedical Research Centre.

## Competing Interests

The Authors declare that there are no competing interests associated with the manuscript.
